# A self-organizing neural network for neuromuscular control

**DOI:** 10.1186/1471-2202-16-S1-P277

**Published:** 2015-12-18

**Authors:** Praveen Shankar, Sharmila Venugopal

**Affiliations:** 1 Department of Mechanical and Aerospace Engineering, California State University Long Beach, CA, USA; 2 Department of Integrative Biology and Physiology, University of California Los Angeles, CA, USA

## 

Adaptive technology holds great promise for sensorimotor rehabilitation in people suffering from spinal cord injury, neuromuscular disease and stroke. With a long-term goal of developing adaptive technology for diagnosis and rehabilitation of neuromuscular dysfunction, we begin the development of a self-organizing neural network (SNN) that compensates for reduced neural drive. We suggest that the self-organizing architecture that adds or deletes nodes online to generate suitable compensatory muscle excitation (Figure 1A) is an apt mechanism to emulate the motor pool behavior of recruitment and de-recruitment of motor units during muscle force generation. Using a virtual muscle [1] resembling the human biceps brachii, we demonstrate the augmentation of neural excitation by the SNN to compensate for abnormal muscle force due to change in the number of motor units.

**Figure 1:**
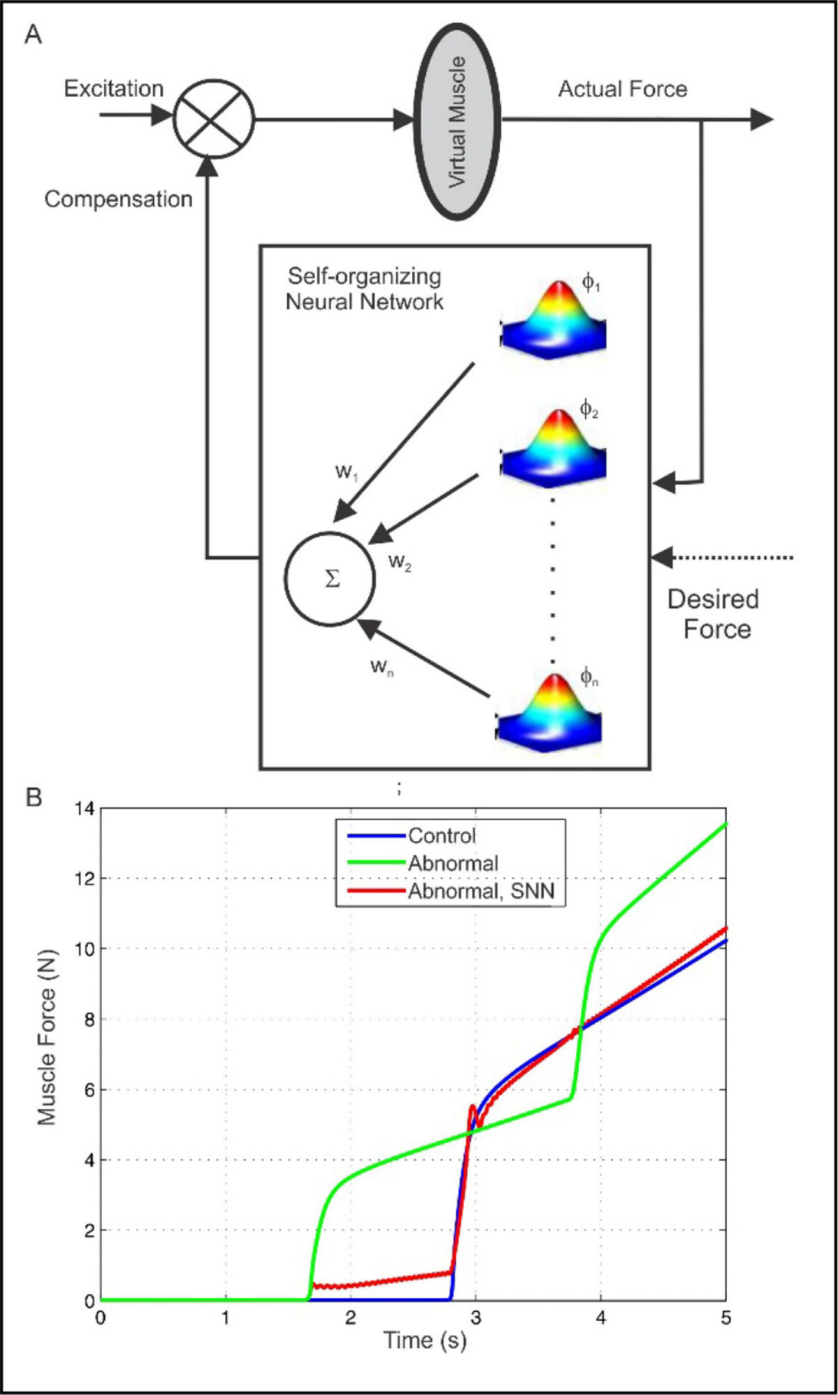
**A. Schematic showing the virtual muscle-SNN system; Φ_1_, Φ_2_, .. Φ_n _are radial basis functions and w_1_, w_2_, ..w_n _are weights for summation**. **B**. Simulation of normal (Slow-Fast motor unit ratio - 2:4), abnormal (Slow-Fast motor unit ratio - 3:3) muscle force and, compensation by SNN.

## References

[B1] Cheng E Brown I Loeb G Virtual muscle: a computational approach to understanding the effects of muscle properties on motor control Journal of Neuroscience Methods 2000 101 117 130 10996372 10.1016/s0165-0270(00)00258-2

